# Chaperone-Mediated Autophagy in Pericytes: A Key Target for the Development of New Treatments against Glioblastoma Progression

**DOI:** 10.3390/ijms23168886

**Published:** 2022-08-10

**Authors:** María Dolores Salinas, Rut Valdor

**Affiliations:** 1 Cell Therapy and Hematopoietic Transplantation Group, Biochemistry, Molecular Biology B and Immunology Department, University of Murcia (UMU), 30120 Murcia, Spain; 2 Unit of Autophagy, Immune Response and Tolerance in Pathologic Processes, Biomedical Research Institute of Murcia-Virgen de la Arrixaca (IMIB), 30120 Murcia, Spain

**Keywords:** glioblastoma, perivascular niche, pericytes, autophagy, chaperone-mediated autophagy, glioblastoma therapy, cell therapy

## Abstract

Glioblastoma (GB) cells physically interact with peritumoral pericytes (PCs) present in the brain microvasculature. These interactions facilitate tumor cells to aberrantly increase and benefit from chaperone-mediated autophagy (CMA) in the PC. GB-induced CMA leads to major changes in PC immunomodulatory phenotypes, which, in turn, support cancer progression. In this review, we focus on the consequences of the GB-induced up-regulation of CMA activity in PCs and evaluate how manipulation of this process could offer new strategies to fight glioblastoma, increasing the availability of treatments for this cancer that escapes conventional therapies. We finally discuss the use of modified PCs unable to increase CMA in response to GB as a cell therapy alternative to minimize undesired off-target effects associated with a generalized CMA inhibition.

## 1. Introduction

### 1.1. Glioblastoma Multiforme

Glioblastoma multiforme (GB) is an aggressive cancer with poor prognosis as it is one of the most difficult cancers to treat [1]. Numerous studies speculate that GB cancer progression may result from a complex multifactorial process implicating different cell types, i.e., astrocytes, neural stem cells, and oligodendrocyte progenitor cells [1,2,3,4,5].

**Figure 1:**
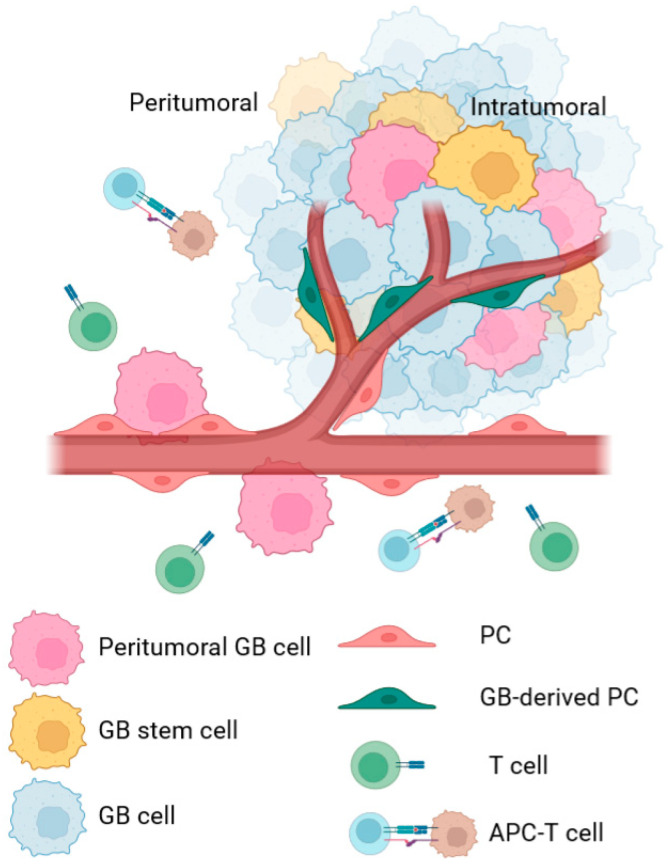
Schematic representation of cell type differences in peritumoral and intratumoral areas of GB cancer. In the peritumoral area, peripheral GB cells interact with peritumoral PCs from pre-existent blood vessels and promote several changes in their phenotype. These changes facilitate processes such as co-optation or peritumoral immune evasion, which help with tumor survival, tumor cell infiltration, and tumor spreading in the brain. In the intratumoral area, most studies identify PCs as cells derived from the tumor, which includes not only regular PCs, but also PCs derived from GSC. These PCs are defined to help in angiogenesis or immune evasion of the tumor. In both areas, the cellular changes are intended to promote tumor survival and growth.

Despite some studies showing that GB is highly angiogenic and hypoxic, this cancer is still poorly characterized [3]. The production of different angiogenic factors, e.g., vascular endothelial growth factor (VEGF), fibroblast growth factor (FGF), and platelet-derived growth factor (PDGF), by tumor cells promotes the development of new vascular vessels, resulting in an intricate network of new blood vessels (Figure 1) [6,7,8]. A common histological hallmark of GB is the presence of glomeruloid microvascular proliferation (GMP) accompanying newly developed blood vessels [9,10]. Nuclei of tumor cells tend to elongate and acquire a palisade shape, forming neat rows surrounding the areas where the tumor undergoes necrosis [8]. In addition, these necrotic areas comprise tumor stem cells and other cells in early stages of differentiation [1,2].

Traits present in the tumor microenvironment (TME) or peritumoral niche have been considered very useful to understand the biology of GB progression as they contribute to tumor immune evasion and support tumor growth [8,11]. GB cells are capable of expressing and secreting immunosuppressive factors (e.g., TGF-β, IL-1, and IL-10), which suppress anti-tumor mechanisms of the surrounding immune cells [8,12,13]. Different immune populations of microglia and macrophages, known as tumor-associated macrophages (TAMs), are regularly found in the peritumoral niche [11,12]. Two types of TAMs have been characterized: M1, which are pro-inflammatory/anti-tumoral; M2, which are anti-inflammatory/pro-tumoral [14]. GB cell phenotypes are driven by the tumor microenvironment, with M2 TAMs being associated with tumor progression [13]. Even B-lymphocytes overexpressing tumor-beneficial immune-inhibitory molecules have been found infiltrating the tumor microenvironment [15]. Thus, GB progression is strongly affected by cells present in the peritumoral niche, and studies involving peritumoral cells have become vitally important. One of these cell types is the pericyte (PC), which resides in the perivascular space of the brain microvessels where GB infiltrates and progresses [16,17]. Here, we explore and discuss the consequences of the GB-driven increase in chaperone-mediated autophagy (CMA) in the PC as a key player in GB progression.

### 1.2. CMA in Cancer

CMA is a selective autophagy pathway that degrades specific proteins in lysosomes. CMA was first observed and thought to be unique to mammalian cells, but recent studies have demonstrated that CMA also occurs in yeasts, birds, and fish [18,19]. Proteins are directly recognized one-by-one by the chaperone heat shock cognate 70 (Hsc70), although other chaperones can be found in the Hsc70-substrate protein complex. Hsc70 recognizes specific KFERQ or KFERQ-like motifs present in the substrate protein, which can be generated via post-transcriptional modifications [20,21]. It is important to note that these motifs are not exclusive for substrate recognition during CMA, as they have been shown to be shared with endosomal microautophagy (e-MI), a different protein degradation pathway that degrades substrates in late endosomes [18].

The only limiting factor for CMA to proceed is the specific receptor located at the lysosomal membrane, the lysosomal-associated membrane protein 2A (LAMP-2A), which is a splice variant of the *lamp2* gene. During CMA, the substrate protein binds to Hsc70 and then to LAMP-2A at the lysosomal membrane to be unfolded, translocated, and degraded within the lysosome with the participation of another chaperone Hsc70, which resides within the lysosome lumen [21,22,23]. CMA activity is highly regulated by cell context and thereby the stimuli that the cell receives, as these modulate the amount of functional LAMP-2A that is mobilized to the lysosomal membrane [21,22]. CMA is an essential quality control system for specific proteins, contributes to the supply of amino acids during long starvation periods, controls proteome remodeling in response to cell context [24,25,26,27], and, when dysregulated, affects cellular functions leading to diseases such as cancer [20,28,29,30,31].

The aging process strongly influences CMA as LAMP-2A levels at the lysosomal membrane becomes less stable at later stages of life. Thus, during aging, a decreased CMA activity causes a lower quality control of proteins and the accumulation of defective proteins, promoting malignant transformation of cells [18,32,33,34].

While physiological CMA levels are needed for cell homeostasis and avoid malignant cell transformation by preventing DNA damage and increasing proteostasis [35], CMA activity becomes pro-oncogenic when dysregulated. Transformed cells abnormally upregulate CMA activity, which degrades different tumor cell survival- and proliferation-inhibitory proteins [32,34,36]. In addition, increased CMA is required to sustain the characteristic Warburg metabolism in tumor cells as it is essential to degrade the majority of glycolytic enzymes and, therefore, to induce anaerobic glycolysis needed for cancer progression [24,37].

Importantly, data from our lab have shown that, in addition to upregulation of LAMP-2A expression and, therefore, increased CMA activity in tumor cells, GB-mediated upregulation of CMA in PCs is also required for tumor growth [17]. Thus, in GB, LAMP-2A has been considered a potential prognosis marker in tumorigenicity [38] as well as a predictor of poor cancer prognosis due to the tumor cell infiltration and progression driven by tumor cell–PC interactions [17,39].

All these implications make CMA an attractive pathway for the development of new therapeutical approaches in GB treatments. However, this is hindered by the current lack of understanding of how CMA is mechanistically regulated and its physiological and pathological specific functions in other cell types. Thus, a better understanding of how CMA works in GB cancer, by studying the tumor niche and the identification of the specific mechanism underlying GB-induced CMA upregulation in PCs, is needed to evaluate the potential of this autophagy pathway, specifically in the PC, as a candidate in the search for new GB treatments.

## 2. CMA in PC during GB Progression

PCs are perivascular stromal cells found in the abluminal wall of venules and can be found in different tissues [40,41]. PCs derive from different embryonic sources and their phenotype depends on the type of tissue and environment in which they are located [42,43,44,45]. This generates a great heterogeneity in PCs, and the context where they are located determines PC function, morphology, and surface markers [46,47].

In the brain, PCs constitute the blood–brain barrier (BBB) along with endothelial cells (ECs) and are involved in various protective functions [41]. Among their functions, PCs can participate in processes with stem cell-like properties, such as angiogenesis [48,49]. They can also perform immune functions acting as macrophages and controlling T-cell migration by secreting different cytokines [50]. PCs can phagocyte and act as antigen-presenting cells, overexpressing major histocompatibility complex (MHC) class I and II molecules, among others, to control T cell responses [51,52].

Thus far, PCs might be considered as tumor attackers, but GB cells can manipulate PC functions, changing their phenotype to promote pro-tumoral instead of anti-tumoral immune responses [17,39,53,54]. Thus, a detailed dissection and a better understanding of the role of CMA in PCs in a healthy brain versus those located in the tumor microenvironment is necessary to develop therapies to counter GB progression mediated by CMA-driven PCs. Tumor cell infiltration and invasion of the brain parenchyma, which provides a proliferative environment, can occur via interaction between tumor cells and parenchyma cells such as PCs [17], creating a large network of microtubes and nanotubes that favors tumor development [16,55]. In addition, different cancer stem cells (CSCs) are also found in the TME, making GB difficult to treat [38,56,57,58]. Thus, the study of the TME, including the perivascular area with peritumoral PCs, is essential to make a good diagnosis of tumor progression.

### 2.1. Intratumoral PC

Intratumoral PCs contribute to angiogenesis and metastasis in GB (Figure 1). In this environment, Type-2 PCs seem to be derived from glioblastoma stem cells (GSCs) and have been shown to participate in tumor growth by promoting angiogenesis [50,59,60]. This angiogenesis is also induced by hypoxia conditions in the TME as PCs are recruited in these conditions via release of VEGF and basic FGF (bFGF) by GB cells [61]. Then, endothelial cells secrete PDGF-BB in response to VEGF and PCs activate the expression of the PDGF receptor in response to bFGF release. As a result, PCs contribute to the blood vessels maturation and vascularization of the tumor [59,61].

Intratumoral PCs are also immunomodulatory, helping tumor cells to evade the anti-tumor immune responses [51,53]. Tumor-derived PCs seem to upregulate PD-L1 expression, a negative regulator of anti-tumor T cell responses, and overexpress the PC marker RGS5, which, together with IL-6, modulates the expression of the adhesion molecule ICAM-1 and promotes T-cell anergy [51]. Furthermore, it has been recently shown how mutations in the epidermal growth factor receptor (EGFR) lead to GB-derived PCs to control pro-tumor responses, supporting previous findings indicating that both vascular and immune TME facilitate tumor growth [54].

### 2.2. Peritumoral PC

In recent years, GB environment and implicated cells and molecules have acquired a special interest. In particular, peritumoral PCs are an attractive target to generate new therapeutical approaches because they are located in small blood vessels of the brain parenchyma, where GB spreads and invades the perivascular space [17,39].

**Figure 2:**
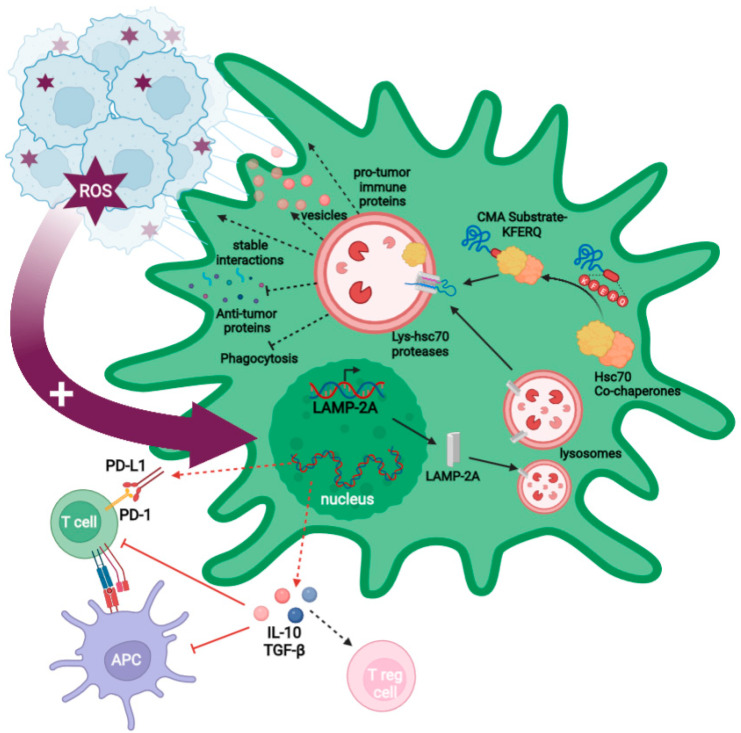
Model of the effects of GB-induced CMA on PCs. GB interaction with PCs promotes a ROS burst that triggers the increased upregulation of CMA in PCs through the expression of LAMP-2A (big garnet arrow). KFERQ motifs in CMA substrate proteins are recognized by the Hsc70 chaperone in a complex with other co-chaperones. This complex binds to substrates and carries them to the lysosomal membrane where they interact with LAMP-2A, and the lysosomal Hsc70 to translocate them into the lysosomal lumen to be degraded. Aberrant induction of CMA by GB leads to changes in the PC proteome, promoting an immunosuppressive function in PCs that exerts a negative regulation on T cells and antigen-presenting cells as a result of changes in gene expression programs that up-regulate the anti-inflammatory cytokines TGF-β and IL-10 (red dashed arrows). The expression of PD-L1 (negative regulator) and the lack of CD80 and CD86 (co-stimulatory molecules) promote regulatory T cell generation (red arrows). GB-induced CMA in PCs also facilitates tumor growth by stabilizing GB–PC interaction and preventing the secretion of anti-tumor proteins and phagocytic ability. In addition, it induces pro-tumor immune molecules and modulates the MSC-like properties such as the increase in the microvesicles secretion with pro-regenerative factors and cytokines that assist tumor proliferation (black dashed arrows).

We have shown that GB interaction with PCs located in peritumoral areas is accompanied by a high expression of the CMA receptor LAMP-2A in both cells. In addition, our data demonstrated that GB cells alter the PC proteome through aberrant degradation of critical proteins by CMA, promoting tumor survival and growth. A transient ROS burst is produced in the PCs following physical interaction with the GB cell, leading to overexpression of LAMP-2A, the only limiting molecule for CMA activity (Figure 2). CMA is then aberrantly upregulated in PCs, consequently affecting the PC phenotype, which promotes stable cell-to-cell interactions that favor tumor cell proliferation, and which induces an immunosuppressive function that impairs anti-tumor immune responses [17,39].

Importantly, peritumoral PCs also seem to participate in the co-optation mechanism of GB, where the tumor does not create new blood vessels (angiogenesis) but kidnaps the existing ones to promote tumor cell infiltration and GB development [62,63].

In addition, a recent RNA-seq study from our lab has shown that the tumor cell–PC interaction affects other functions in the PCs that might play important roles in GB progression. Supporting previous data, our study has revealed that GB-induced CMA in PCs promotes changes in gene expression pathways related to angiogenesis, cell adhesion, and immune and inflammatory functions of PCs. In fact, one of the most affected gene pathways in PCs interacting with GB was related to the phagosome, allowing us to identify the phagocytic function of PCs affected by CMA [39].

In conclusion, GB cell–PC interaction leads to aberrant increased CMA activity in PCs (peritumoral and probably intratumoral), leading to major proteome changes in these cells. These promote the transformation of PCs from pro-inflammatory to anti-inflammatory cells and change other functions that affect the TME. As a consequence, the tumor escapes from anti-tumor immune responses and progresses (Figure 1). Thus, GB-induced CMA in PCs might be considered as a candidate therapeutical target to treat GB cancer.

### 2.3. GB-Induced CMA in PC

#### 2.3.1. GB-Induced CMA in PC Is Required for GB Immune Evasion

One of the most important CMA-mediated changes in PCs following interaction with GB is the acquisition of an anti-inflammatory phenotype. GB-conditioned PCs increase the secretion of anti-inflammatory cytokines (e.g., IL-10 o TGF-β) (Figure 2), while GB cells prevent an increased secretion of granulocyte–macrophage colony-stimulating factor (GM-CSF) related to their survival [17,53].

GB-induced CMA is also responsible for the reduced expression of co-stimulatory molecules and MHC-II surface molecules in PCs, affecting antigen presentation and, therefore, impairing T cell activation, which, in turn, fails the anti-tumor T cells responses (Figure 2) [17,30,53]. Moreover, these changes have been recently corroborated by a transcriptomic study with CMA-deficient PCs, showing that inflammatory and immune gene expression pathways are affected by GB-induced CMA. These data revealed that PCs prevent GB elimination by their phagocytic capacity and the secretion of anti-tumor immune molecules, via GB-induced CMA [39]. In addition, these data showed that GB-induced CMA in PCs also leads to the secretion of molecules that can promote pro-tumor immune responses, such as gelsolin, periostin, and osteopontin, supporting previous data showing the contribution of the PC secretome to GB immune evasion [17,39].

#### 2.3.2. GB-Induced CMA Upregulation in PC Is Required for Stable PC–GB Interactions

GB–PC interactions lead to a functional network of connections that facilitate nutrient support and GB proliferation [16,17,55]. GB-induced CMA upregulation is necessary to create the stable interactions between GB cells and PCs that maintain tumor cell survival and tumor growth. Those stable interactions are formed by nanotubes originated in PCs to GB cells and the regulation of adhesion protein expression (e.g., occludin), which is also dependent on GB-induced CMA [17,39].

Using a GB mouse model with grafted CMA-deficient PCs, we have previously shown a decrease in GB–PC interactions and impaired tumor formation when mice were infiltrated with GB cells [17]. The characterization of cell-to-cell interactions in CMA-deficient PCs showed an absence of stable interactions and a subsequent reduction in GB cell survival. By contrast, mice grafted with control GB-conditioned PCs showed stable PC–GB interactions and tumor cell proliferation. Further electron microscopy analyses in PCs revealed a microvesicle secretion pattern dependent on GB-induced CMA. Interestingly, this vesicle secretion was mainly observed in the zone where the GB cell interacts with the PC [17].

#### 2.3.3. GB-Induced CMA in PC Is Required to Modulate PC Secretome and PC MSC-like Properties That Favor Tumor Progression

GB-induced CMA in PCs protects GB from a toxic PC secretome by inhibiting secretion of antitumor effectors (e.g., sparc, antithrombin, lumican, and vitamin D), which could be capable of destabilizing and inhibiting GB–PC interactions, causing GB cell death [17,39]. By contrast, GB-induced CMA in PCs induces a secretome with high levels of proteins that can promote tumor angiogenesis and facilitate pro-tumor immune responses (e.g., angiotensin, gelsolin, periostin, and osteopontin) [39].

Furthermore, GB-induced CMA in PCs affects their mesenchymal properties, facilitating tumor angiogenesis and progression. The expression of different angiogenic, angiotrophic, or anti-inflammatory factors changes when PCs interact with GB cells [17]. Two of these factors are IL-6, a cytokine with regenerative properties, and the angiogenic factor VEGF, both highly secreted in GB-conditioned PCs [53]. In addition, PC proliferation is reduced in response to GB-induced CMA, correlating with a loss of mitochondrial mass and the modulation of markers associated with mesenchymal properties (e.g., Sca-1, CD105, and CD90) [17].

## 3. Possible Therapeutic Approaches Based on CMA Inhibition in PC to Counter GB Progression

GB is one of the few cancers in which conventional therapies, i.e., surgery, radiotherapy, and pharmacotherapy with temozolomide (TMZ), do not increase survival of the patients [64]. As previously discussed, GB has a very heterogeneous niche, which provides therapeutic resistance and limits the availability of effective therapies. The current chemotherapeutic for GB is usually TMZ, which is used in combination with irradiation to prevent resistance [64]. Interestingly, TMZ seems to induce expression of LAMP-2A and, therefore, CMA activity in human tumors [65], which might explain why tumor cells become resistant and PCs are, at least in part, responsible for the increased resistance [66]. As dose intensification fails to boost effectiveness, this treatment only prolongs the median survival of patients for 20 months after diagnosis [64]. Other alternative approaches focused on CAR-T- [67] and NK cell-targeted therapies [68] or target-specific mutations and tumor metabolism [64] are still under study [36].

Thus, this situation has encouraged the urgency of finding new targets that can prevent GB development and reappearance. This is the case of CMA in PCs, a key target for the development of new treatments against tumor progression [17,39].

### 3.1. Possible Side-Effects Following Therapy That Promotes a Generalized CMA Inhibition

**Figure 3:**
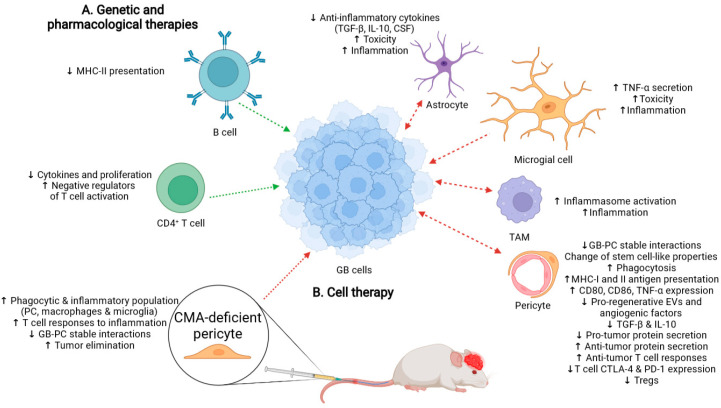
Possible immune responses after CMA inhibition therapies to prevent GB progression. In immune and brain-resident cells, CMA may be altered in different ways in response to GB, and its inhibition might favor tumor progression (green arrows) or promote anti-tumor activity (red arrows). Thus, (**A**) future gene and pharmacological therapies against CMA should take into account that cells of the adaptive immune response, such as CD4^+^ T cells and B cells, require CMA to develop their anti-tumor responses. CD4^+^ T cells need CMA for proper activation, including cytokine release and proliferation through degradation of negative regulators of T cell activation. B cells also need CMA for maintaining antigen presentation. However, inhibition of CMA in TME cells could have an anti-tumor effect. For instance, CMA decreases the inflammasome-mediated responses of TAMs and TNF-α secretion in microglial cells. Astrocytes also require CMA to acquire an anti-inflammatory phenotype dependent on physical contact with tumor cells. Thus, CMA inhibition would maintain an inflammatory phenotype that would aid tumor elimination. In addition, aberrant GB-induced CMA upregulation in PCs promotes cancer cell survival and progression through cell–cell stable interactions, contributing to the immunosuppressive peritumoral niche with the secretion of pro-tumoral proteins, angiogenesis promotion, and pro-tumoral macrophages recruitment. Therefore, CMA ablation in PCs would prevent GB–PC interaction, maintaining a pro-inflammatory phenotype, among other anti-tumor changes, that reduce tumor progression and promote tumor clearance. On the other hand, (**B**) CMA-deficient PCs can be used as an alternative therapy that can avoid possible side-effects of CMA inhibition in healthy and GB-conditioned cells. Modified PCs are able to inhibit GB tumor growth and induce tumor clearance preventing PC–GB interactions. They could no longer be modulated by GB cells and present a pro-inflammatory function that prevents tumor growth and helps tumor elimination by stimulation of the anti-tumor immune responses. This effect is partly due to the secretion of pro-tumor molecules (lumican, vitamin D, among others), increased phagocytosis ability, and antigen presentation. Hence, CMA-deficient PCs can stimulate TME cells, increasing phagocytic and inflammatory populations (macrophages, PCs, and microglia) and promoting T cell inflammatory responses.

In GB cancer, not only is CMA induced in the tumor cell, but it is also aberrantly upregulated in PCs as a consequence of GB–PC interactions (Figure 2) [17]. Thus, inhibition of CMA not only in the tumor cell, but also in PCs (Figure 3A), may be considered as a new strategy in the search for novel therapies against GB. However, drugs that specifically inhibit CMA do not exist, and only a few compounds such as HQ [32], a phosphopeptide [69], vitamin E [70], p38 MAPK inhibitors, anisomycin, or cycloheximide [71] have been shown to downregulate CMA activity. As these CMA modulators have also other targets [37] or can only affect a certain cell type [69,72], gene therapy directed to block only LAMP-2A expression (e.g., with the CRISPR-Cas system or with siRNA) could provide a much greater specificity as an anti-cancer strategy [32,36]. However, it is still far from being tested as a treatment in clinical trials for GB as it might affect the anti-tumor immune function of some cell populations different to PCs [36].

On the other hand, it is essential to consider that CMA is required for the cell response to stress [32] and some specific immune functions in different cell types that are critical to counter GB progression [31,36,73]. CMA activity naturally decreases during aging, and its failure might worsen the outcome of tumor prevention by anti-tumor immune responses [73,74]. In addition, macroautophagy has been shown to be induced when CMA is blocked, allowing cells to survive under normal conditions when they lose their tolerance to particular stressors [75]. Although these compensation effects do not occur in cancer cells [76], this could mean that premature generalized inhibition of CMA as a therapy in peritumoral cells with upregulated CMA activity might lead to unwanted side-effects in nontarget cells (Figure 3A).

Even though the role of CMA in CD8^+^ T cells has not been described yet, CD4^+^ T cells are known to upregulate CMA to be efficiently activated and degrade ITCH and RCAN1, two negative regulators of TCR signaling [30]. Something similar has been described for some antigen-presenting cells such as B cells, which need LAMP-2A to be able to internalize and process autoantigens for MHC-II presentation and activate T cells [77,78]. Thus, the deficiency of CMA also compromises immunization and pathogen infection [21,30,79]. Consequently, therapies that target CMA should be evaluated to ensure that they do not cause immunosuppressive responses (Figure 3A).

By contrast, upregulation of CMA by cancer cells does not only affect PCs, but also some types of peritumoral cells such as TAMs (Figure 3A) [36]. TAMs also present tumor cell-mediated LAMP-2A upregulation that supports tumor growth [80]. TAMs seem to produce IL-17 to activate CMA and become tolerant to apoptosis-inducing treatments in cancer [81]. Hence, normal macrophages seem to need the inhibition of CMA activity to promote inflammatory responses through inflammasome activation (Figure 3A) [29].

Interestingly, CMA upregulation in microglial cells and astrocytes also prevents brain inflammation and toxicity, resulting in neurodegeneration [82]. CMA is needed by glial cells, which occurs in neurons to avoid accumulation of poorly processed proteins that cause severe brain damage [22,34]. Microglia can degrade IKKβ and reduces TNF-α expression levels through CMA in response to a specific stimulus [83]. Thus, CMA inhibition might increase inflammation in glial cells around the tumor, although it might also cause neuronal damage and cellular toxicity.

CMA also regulates innate immunity responses through degradation of stimulator of interferon genes protein (STING), which accumulates during inflammation stages against viral infections [84] and is degraded in the later stages to stop inflammation, which occurs in cancer [21,85,86].

In summary, generalized CMA blockage could help clear the tumor and prevent its progression, but it might have associated negative consequences as CMA is required for the normal activity of cells to maintain brain homeostasis. This inhibition could even promote aggressive brain inflammation and affect the anti-tumor T cell responses needed for countering GB progression (Figure 3A).

### 3.2. Cell Therapy with CMA-Deficient PC as an Alternative to Treat GB

We have recently shown a possible therapeutic approach to inhibit GB progression by using exogenous PCs with inhibited CMA. Our work shows that cell therapy using CMA-deficient PCs is enough to inhibit tumor growth and promote GB cell elimination following anti-tumor immune responses (Figure 3B) [39]. As PCs lacking CMA prevent GB interactions that lead to immunosuppressive function [17], we hypothesized that administration of CMA-deficient PCs should lead to an inhibition of tumor growth and a lower infiltration capacity. Then, we tested different therapeutical strategies such as intracranial therapy compared to intravenous administration of exofucosylated PCs with and without CMA. Exofucosylation is based on the transient conversion of native CD44 into the sialofucosylated glycoform, known as hematopoietic cell E-/L-selectin ligand (HCELL) [87,88], which can be used as a cell-engineering strategy to deliver cells into inflammation areas. As GB cells overexpress E-selectin [89], HCELL is able to strongly and specifically bind to them [53,90]. Our GB mouse model showed that CMA-deficient PCs injected intravenously were able to reach the tumor brain parenchyma, accumulate in peritumoral areas around blood vessels of the brain cortex, and generate a pro-inflammatory anti-tumor response. Compared with intracranial cell therapy, which also works efficiently [39], this therapy consisting of intravenous administration of modified PCs can be efficient to prevent GB progression and also eliminate the tumor cells while being far less invasive [17,53]. This last strategy also seems to activate different phagocytic cell populations that may promote the anti-tumor immune responses (Figure 3B). The detection of activated immune cells in the brain parenchyma coming from circulation following intracranial cell therapy might indicate a more aggressive strategy [52,91,92]. Thus, intravenous administration is less invasive and aggressive, and it could be used in combination with other treatments. With our work, we demonstrated that CMA in PCs is a key target to develop alternative treatments against GB cancer, which may prevent side-effects of the CMA inhibition in other cell types, caused by pharmacological or genetic approaches.

## 4. Conclusions and Future Perspectives

GB is a very aggressive tumor that develops in a vital tissue with poor prognosis due to the inefficacy of current anti-tumor treatments. Thus, new therapies that can stop its growth and achieve a cure must be developed. Direct therapy against this glioma is very complicated due to GB’s intrinsic cellular heterogeneity and the potential adverse effects that this intervention might have on peritumoral cells. Pharmacological manipulation of CMA might be used as a specific therapy against GB growth. Inhibition of CMA would directly impact GB cells as well as other tumor-related cell types such as PCs, which help the tumor spreading in the brain. However, specific inhibitors against LAMP-2A or CMA modulators have not been found or demonstrated to work. Gene therapy directed to modulate CMA in PCs specifically should be cautiously considered as an alternative as physiological CMA in PCs is essential to maintain healthy brain homeostasis.

Tumor cells wickedly manipulate and increase CMA in PCs to benefit from changes in the TME, and this can be seen as an opportunity to develop new pharmacological therapies countering this process to treat GB. The development of such treatments might also be useful in the treatment of other highly vascularized cancer types where PCs show a pro-tumoral regulatory role [92]. Generalized CMA blockage in peritumoral brain cells (including PCs and probably other immune cells) should lead to a pro-inflammatory niche capable of preventing tumor progression and promoting anti-tumor immune responses to eliminate GB cells. However, T cells need CMA activity for a proper activation and function and, therefore, the immunomodulation that CMA inhibition could have in T cell responses will require further study with combined therapies to boost their anti-tumor immune responses. Moreover, generalized CMA inhibition in brain-resident immune cells could lead to undesired brain inflammation. Cell therapy with modified PCs without CMA might provide a solution to eliminate tumor cells and prevent possible undesired side-effects of CMA inhibition in the brain. Importantly, it is possible to deliver CMA-deficient PCs with an anti-tumor immune function to GB-affected brain areas by intravenous via and using a strategy for cell therapy known as “exofucosylation”. In conclusion, CMA-deficient PCs are very attractive cells to develop new candidate treatments to fight GB cancer. Cell therapy with CMA-modified PCs might be used in combination with current therapies or other immunotherapies to reduce brain metastasis and recurrence after surgery, and thus, to not only treat GB, but even other highly vascularized cancers. A next step further would implicate the use of therapies to modulate GB-induced CMA upregulation in PCs, as these cells would be able to carry on any housekeeping function implicating physiological CMA but would be refractory to GB manipulations.
